# Biodegradable Carbohydrate-Based Films for Packaging Agricultural Products—A Review

**DOI:** 10.3390/polym17101325

**Published:** 2025-05-13

**Authors:** Kshanaprava Dhalsamant, Asutosh Dalai, Falguni Pattnaik, Bishnu Acharya

**Affiliations:** Department of Chemical and Biological Engineering, University of Saskatchewan, Saskatoon, SK S7N 5A9, Canada; shreeprava@gmail.com (K.D.); asd268@usask.ca (A.D.); pattnaikfalguni1994@gmail.com (F.P.)

**Keywords:** biodegradable packaging, carbohydrate-based film, polysaccharides, agricultural commodities, sustainable development

## Abstract

Carbohydrate-based biodegradable films offer an eco-friendly alternative to conventional petroleum-derived packaging for agricultural commodities. Derived from renewable polysaccharides such as starch, cellulose, chitosan, pectin, alginate, pullulan, and xanthan gum, these films exhibit favorable biodegradability, film-forming ability, and compatibility with food systems. This review presents a comprehensive analysis of recent developments in the preparation, functionalization, and application of these polysaccharide-based films for agricultural packaging. Emphasis is placed on emerging fabrication techniques, including electrospinning, extrusion, and layer-by-layer assembly, which have significantly enhanced the mechanical, barrier, and antimicrobial properties of these materials. Furthermore, the incorporation of active compounds such as antioxidants and antimicrobials has improved the performance and shelf-life of packaged goods. Despite notable advancements, key limitations such as moisture sensitivity, poor mechanical durability, and high production costs persist. Strategies including polymer blending, nanofiller incorporation, and surface modification are explored as potential solutions. The applicability of these films in packaging fruits, vegetables, dairy, grains, and meat products is also discussed. By assessing current progress and future prospects, this review underscores the importance of carbohydrate-based films in promoting sustainable agricultural packaging systems, reducing environmental impact through the advancement of circular bioeconomy principles and sustainable development.

## 1. Introduction

Packaging is challenging in the agricultural industry as it ensures secure transportation, storage, and promotion of goods from the farm to the customer. Efficient packaging preserves the quality and safety of agricultural products, prolonging their shelf life and minimizing post-harvest losses [1,2]. Additionally, it renders crucial roles such as conveying information, improving product visibility, and streamlining handling and distribution processes [3]. Plastic production and consumption have been increasing exponentially in recent decades: global plastic production reached approximately 460 million tonnes in 2022, with nearly 40% allocated to packaging applications [4]. Plastic is one of the most widely used materials for packaging due to its versatility, durability, and low cost [5,6]. Although conventional plastic packaging offers advantages, it also poses substantial environmental obstacles. Plastics, obtained from finite fossil fuel resources, contribute to the contamination and deterioration of the environment. A significant portion of plastic waste ends up in the environment, causing pollution in oceans, landfills, and ecosystems. The United Nations Environment Programme (UNEP) reports that more than 350 million tonnes of plastic waste are generated annually worldwide, of which only 9% is recycled, 19% is incinerated, and the remaining 72% ends up in landfills or the environment [7,8]. Disposing of plastic garbage by incineration or landfilling worsens environmental issues by increasing greenhouse gas emissions and causing landfill overflow [9,10]. They exhibit a high degree of persistence in the environment, resulting in problems such as marine pollution, soil contamination, and adverse effects on wildlife [11,12]. Tiny plastic particles, known as microplastics, are increasingly found in various environments, including water bodies and human tissues [13,14]. Many countries and organizations are implementing measures to manage the usage of plastic [15]. Recycling plastic can be complex due to contamination, varying types, and limited infrastructure [16]. In recent years, legislative actions across the globe have increasingly targeted the reduction of plastic waste, thereby catalyzing the development of biodegradable and compostable packaging alternatives. The European Union’s Directive (EU) 2019/904, aimed at curbing the impact of single-use plastics, mandates the gradual phasing out of non-compostable plastic materials and promotes circular economy principles [17]. India’s Plastic Waste Management (Amendment) Rules, 2022, enforce strict bans on specified single-use plastics and promote extended producer responsibility (EPR) in packaging systems [18]. Similar regulations are being implemented in Canada, Australia, and several U.S. states, with a focus on sustainable material substitution, recyclability, and biodegradability. Simultaneously, there is a growing demand from consumers for packaging that is safe, compostable, non-toxic, and derived from renewable resources. Environmental awareness, coupled with concern over microplastic pollution and health risks, has shifted consumer preferences toward bio-based packaging solutions [14]. Market analyses indicate that brands adopting eco-friendly packaging are increasingly favored by sustainability-conscious consumers [5]. Together, these regulatory and societal pressures have intensified the search for biodegradable alternatives such as polysaccharide-based films, reinforcing their significance as a focal area for both research and industrial innovation [5,14]. Biodegradable packaging is specifically engineered to decompose organically through the activity of microorganisms, hence minimizing its impact on the environment [19,20]. These materials provide numerous advantages, including sustainability (derived from renewable resources, they decrease reliance on fossil fuels), decreased pollution (undergo decomposition into natural components, hence limiting pollution and waste), and compostability (materials can undergo composting, which enhances the quality of soil and contributes to the circular economy by completing the recycling process) [19].

**Figure 1:**
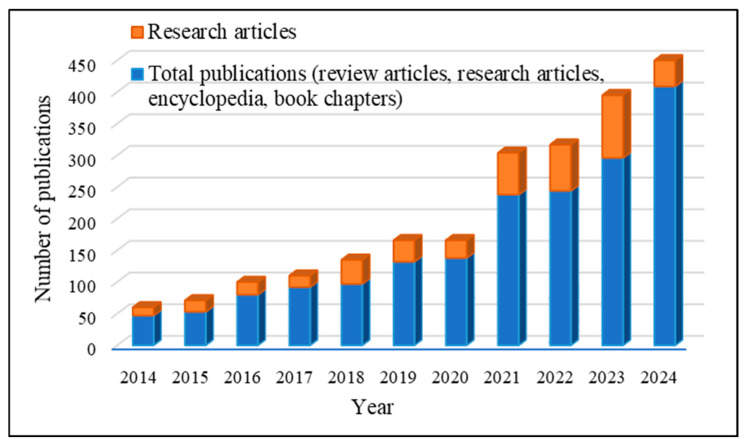
Number of publications on biodegradable film for packaging agricultural commodities, including review articles, research articles, encyclopedia, and book chapters in the last decade (2014–2024); Source: Scopus, Web of Science, and Google Scholar.

**Figure 2:**
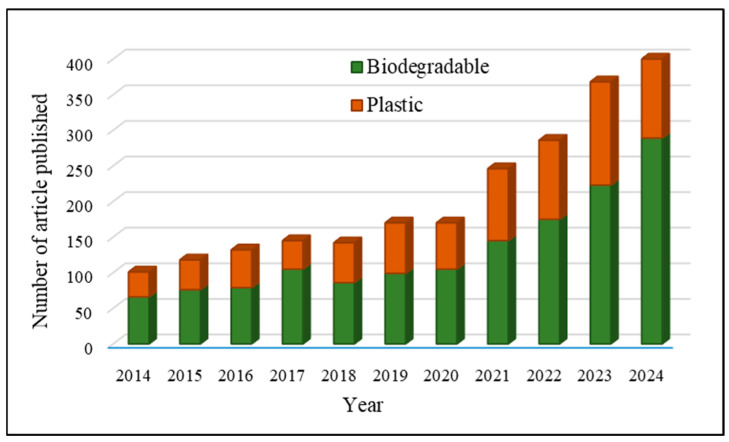
Academic publications on biodegradable film and plastic film in the last decade (2014–2024); Source: Scopus, Web of Science, and Google Scholar.

Figure 1 provides a comprehensive summary of the body of literature, including review papers, research articles, encyclopedias, and book chapters, produced in the past 10 years on the biodegradable film for packaging agricultural commodities. The graph’s trend indicates the level of interest in the subject exhibited over the past decade and predicts an increasing demand in the future. Similarly, Figure 2 displays scholarly articles on biodegradable films and plastic films throughout the past 11 years.

There are several types of carbohydrate-based biodegradable films, including films derived from cellulose, starch, polylactic acid (PLA), polyhydroxyalkanoates (PHA), chitosan, pectin, and algal polysaccharides. Cellulose-based films are derived from cellulose, the primary structural component of plant cell walls, usually from wood, cotton, or agricultural residues [21]. Cellulose and its derivatives offer superior mechanical strength and gas-barrier properties, but the insolubility of native cellulose necessitates chemical modification or derivatization for film formation [22]. Starch-based films are derived from starch, which is abundant in plants like corn, potatoes, and cassava [23]. Starch, due to its abundant availability and low cost, is extensively used; its amylose content contributes to its film-forming capability, although its high-water sensitivity limits performance under humid conditions [23]. PLA-based films are derived from the fermentation of renewable resources like corn starch or sugarcane to produce lactic acid, which is then polymerized into PLA [24]. PHA-based films are produced by bacterial fermentation of sugars or lipids, making PHA a bio-based and biodegradable polymer [25]. Chitosan-based films are derived from chitin, found in the exoskeletons of crustaceans (e.g., shrimp and crabs) and insects [26]. It is especially valued for its intrinsic antimicrobial activity and film transparency, making it ideal for perishable food packaging, though it requires acidic solvents for processing. Pectin-based films are derived from pectin, a naturally occurring polysaccharide found in the cell walls of fruits like apples and citrus, and produce transparent, antioxidant-rich films suitable for oil-containing foods, but they typically require cross-linkers or blending to enhance their mechanical strength and water resistance [27]. Algal-based films are derived from seaweed or algae, rich in polysaccharides like agar, carrageenan, or alginate [28]. Algal polysaccharides, especially alginate, form strong and flexible films via ionic cross-linking with divalent cations such as calcium; they are particularly advantageous for applications requiring breathable, edible, or moisture-retaining films. In this way, carbohydrate-based films have become a promising choice for packaging among different biodegradable materials as they are fabricated from the natural polymer, obtained from plant biomass, and demonstrate exceptional abilities to create films [25,26,27,28,29,30]. These films exhibit biodegradability, as well as desirable attributes including flexibility, transparency, and mechanical strength [19]. Additionally, films made from different biopolymers can be customized to improve their ability to block moisture and gases, which makes them well-suited for packing various agricultural products [19,20].

While biodegradable packaging materials offer an environmentally preferable alternative to conventional plastics, it is important to acknowledge that they are not without limitations [31]. The production of biopolymer-based films often involves energy-intensive processes, high costs, and resource constraints. Additionally, improper disposal or lack of industrial composting facilities may limit their environmental benefits [21]. Therefore, addressing the challenges of material sourcing, processing efficiency, and end-of-life management is essential for truly sustainable outcomes. A rational and optimized use of both petroleum-based and bio-based materials, based on application needs, functionality, and environmental impact, is necessary to develop holistic packaging solutions [16,21]. Integrating circular economic principles and sustainability assessments into material design and selection can facilitate the transition toward a more resource-efficient packaging sector [20].

This paper presents a comprehensive review of the potential of carbohydrate-based biodegradable films for packaging applications in the agricultural sector. It aims to: (i) analyze the physicochemical characteristics and fabrication techniques of films derived from various carbohydrate sources, including cellulose, starch, chitosan, and other polysaccharides; (ii) evaluate their functional performance and suitability in agricultural packaging systems; (iii) highlight their environmental, economic, and technological advantages over conventional plastic packaging; (iv) identify current challenges, limitations, and barriers to widespread adoption; and (v) explore recent innovations and future research directions in the development of carbohydrate-based films. Moreover, this review primarily focuses on the application of carbohydrate-based biodegradable films in packaging a range of agricultural commodities, including fresh produce (fruits and vegetables), cereals and grains, dairy products, meat and poultry, oil-rich foods, and dry food items. These categories were selected based on their high perishability, sensitivity to oxygen and moisture, and the growing demand for sustainable packaging alternatives in these segments. To ensure a comprehensive and balanced perspective, the literature was sourced from reputable scientific databases such as Scopus, Web of Science, and Google Scholar. The search strategy included keywords such as “carbohydrate-based films”, “biodegradable packaging”, “polysaccharides”, and “agricultural commodities”. The review included peer-reviewed research articles, review papers, and relevant book chapters published primarily between 2014 and 2024, with a preference for studies emphasizing fabrication methods, functional performance, and sustainability. Studies lacking experimental validation or unrelated to food/agricultural applications were excluded to maintain the relevance of the discussion. This methodological framework ensures that the review provides a focused and up-to-date synthesis of advancements in biodegradable films made from renewable carbohydrates.

## 2. An Overview of the Different Types of Carbohydrates and Their Sources

### 2.1. Pectin

The structural polysaccharide that is mostly produced during the early development phase of the primary cell wall is pectin, a complex heteropolysaccharide that exists naturally in plant cell walls. Equally abundant in young and fragile tissues is this component [32]. Various types of pectin are defined by the side chains that bind the galacturonic acid residues together; these include Xylogalacturonan (XGA), Homogalacturonan (HG), Rhamnogalacturonan I (RG-I), Rhamnogalacturonan II (RG-II), and Apiogalacturonan (AP). The extraction method is also significant if the structure, composition, and properties imposed by this complex biopolymer are not to be ruined. Pectin extraction methods can be broadly divided into two sorts: traditional and innovative. Traditional methods are acid-mediated, high-temperature extractions that have various environmental impacts. By contrast, innovative methods include ultrasound-assisted extraction, microwave-assisted extraction, and enzyme-assisted extraction, which are considered greener, more eco-friendly ways to recover this valuable raw material [33].

In the food industry, pectin is widely employed as a thickener, emulsifier, and adhesive agent. It also serves as the basis of a new type of biomaterial utilized in the production of bio-sustainable packaging films and coatings through its superb physicochemical properties. Active food packaging applications typically use pectin-based films and coatings [34]. The traditional method of pectin extraction is acid-mediated hot extraction. Generally, that means treating a pectin source material with a strong acid (hydrochloric acid, typically) at high temperatures (75–100 °C) for as long as 3 h [35]. The extraction duration, particle size, solid–liquid ratio, temperature, and pH all have significant impacts on the pectin quality.

Recent advancements have led to the development of many non-traditional extraction technologies, including ultrasound-assisted, microwave-assisted, and enzyme-assisted extraction [36]. These approaches are regarded as more ecologically sustainable options. Ultrasound-assisted extraction employs ultrasonic vibrations or sound waves to disrupt the cell walls of the pectin source material, hence enhancing solvent penetration and mass transfer kinetics [36,37]. It necessitates reduced solvent usage, diminished energy consumption, and enhanced yields with shorter extraction durations. Microwave-assisted extraction utilizes microwave radiation to create heat in the presence of dielectric materials. This thermal impact enhances the extraction process by augmenting the diffusibility of substances between the sample and the solvent [36,37].

The enzyme extraction technique uses enzymes that digest the cell walls of plants so as to liberate pectin and reduce the necessary amount of extraction time. With different enzymes extracting pectin, their action method varies. However, different ways of extracting pectin were found to significantly affect both its structure and properties. According to [38], the molecular weights of pectins extracted using ultrasound-assisted technologies were 386 kDa, microwave extraction was 264 kDa, and conventional acid extraction was 263 kDa. The traditional extraction approach yielded an elevated level of esterification (DE) in the pectin (84%), surpassing the results achieved by the microwave-assisted (74%) and ultrasound-assisted (77%) methods [37].

Edible films made from bio-based materials, such as pectin, contain a number of antioxidant characteristics, are biocompatible, and can act as gelling agents. Films made of pectin have found extensive application as a biodegradable packaging material for a variety of foods, including soybean oil, cheese, pork, and fish [35]. Edible films were coated with pectin-based functional compositions to extend their shelf life [36]. Research has shown that pectin-based coatings can delay the ultimate demise of the majority of forms of methanogens, which means that they can extend the shelf life of food products [36].

### 2.2. Starch

Starch, a carbohydrate-based biopolymer, is widely utilized in food packaging applications. The advancement of technology enabling starch utilization has led to reduced production costs and an abundant supply. However, starch-derived plastic materials are distinct from conventional plastics. Unlike traditional plastics, starch-based materials exhibit excellent biodegradability without the necessity for costly additives, and they are readily available and applicable without requiring industrial detergent treatments typically needed for waste materials. Nevertheless, starch-based films suffer from poor mechanical properties, relatively low water resistance, and significant vulnerability to moisture, particularly at low temperatures [39]. To address these limitations, thermoplastic starch is often combined with various nanofillers to enhance its properties. Starch is sourced from a range of plants, including rice, wheat, corn, and tuber crops such as potatoes. Structurally, starch is composed of two types of microstructures: a linear structure (amylose) and a branched structure (amylopectin) [40]. Furthermore, plasticizers are incorporated to control the brittleness of starch-based films. Plasticizers are defined as agents that induce orientation, partial transition, and sudden formation of a smooth elastomeric polymer phase. Their addition generally improves mechanical properties; although the tensile stress–strain behavior is somewhat weakened, leading to a lower tensile strain, they concurrently reduce hardness and density, decrease viscosity, enhance polymer chain flexibility through constructive changes, and improve resistance to fracture [41].

Starch can be extracted by several methods. Following extraction, the material produced is a starch-based biodegradable material made by processing with heat to yield a filmogenic solution [42]. The preferred choice for this use is starch, which has more amylose content because it has a larger crystalline domain, which provides it with better mechanical strength. Starch-based films have been used to pack a variety of food products such as chicken fillets, tomatoes, and bread. The films are found to reduce the loss of water to keep these products moisture less and also to inhibit the growth of microbes an increase in shelf life of both these properties [43].

One of the major remaining problems with starch-based films is that they cannot be induced to show any high or even medium-level mechanical strain and have poor water vapor permeability. Poor mechanical behaviors are evaluated using tensile tests. In the case of such tests, they include tensile strength and strain at necking. In terms of high-water vapor permeability, the problem lies in hydrophilicity [44]. Hydrophilicity is a property that arises from the polar characteristic of the hydroxyl groups of starch and is, therefore, seen as one of the important factors influencing starch-based biodegradable films destined for food packing sites [45]. Further research needed on the possibility of using starch films for wrapping agricultural produce is outlined in this work. We need to improve the properties of these films and develop new methods for their production.

### 2.3. Cellulose

Cellulose, a naturally occurring and abundantly available biopolymer, demonstrates considerable potential for application in biodegradable films intended for agricultural packaging. It is highly valued due to its excellent film-forming properties, strong mechanical characteristics, and significant water resistance, which support its versatility across various packaging applications. Despite its beneficial properties, the insoluble crystalline structure of cellulose renders it insoluble in common solvents, necessitating its conversion into water-soluble derivatives that are more amenable to film formation and processing. Derivatives such as methylcellulose, carboxymethyl cellulose, and hydroxypropyl methylcellulose are commonly utilized to fabricate biodegradable films [22,46]. These films offer effective barrier properties against oxygen transmission and aroma permeability. However, their water vapor barrier properties remain suboptimal. To enhance the performance of cellulose-based films, numerous additives, such as hydrophobic compounds and nanomaterials, are increasingly explored as alternatives or reinforcements to cellulose and its derivatives [46]. These cellulose derivatives are also being applied as reinforcing agents in combination with other biodegradable polymers to improve mechanical robustness and thermal stability [46]. Although cellulose derivatives are naturally used as gelling agents, edible films composed of their native molecular sizes may exhibit inadequate physical and thermal properties, thereby limiting their use in certain food packaging scenarios. The incorporation of nanoscale cellulose, which possesses high tensile strength and excellent adhesion properties, has emerged as a promising solution to these challenges. In its natural form, cellulose is indigestible to humans [22,47]. Nonetheless, recent advancements have facilitated the transformation of raw cellulose into edible materials, broadening their applicability in food contact applications and packaging systems [46]. This progress has paved the way for the development of novel biomedical and food-grade materials based on cellulose. In conclusion, cellulose and its derivatives present a highly promising alternative to petroleum-derived materials for the development of biodegradable films. Their widespread availability, low cost, and biodegradability make them suitable candidates for sustainable packaging [47]. Ongoing research efforts are focused on further enhancing the functional properties of cellulose-based films to better accommodate the specific requirements of various agricultural commodities.

### 2.4. Chitosan

Chitosan is a linear polysaccharide composed of β-(1→4)-linked D-glucosamine (the deacetylated unit) and N-acetyl-D-glucosamine (the acetylated unit). It is typically derived from chitin, which is found in the exoskeletons of crustaceans such as shrimp, through a deacetylation process involving treatment with an alkaline solution, most commonly sodium hydroxide [48]. Chitosan possesses a range of intrinsic properties that contribute to its broad utility across various fields. It is biocompatible, indicating that it does not elicit toxicity in living tissues, and it is biodegradable, allowing it to be decomposed by environmental microorganisms. Chitosan is widely recognized for its intrinsic antimicrobial activity in solution form, primarily attributed to the interaction of its protonated amino groups with microbial cell membranes [49]. However, the literature evidence suggests that this antimicrobial efficacy is significantly diminished when chitosan is incorporated into solid film matrices [50]. This reduction is due to the limited mobility of chitosan chains and decreased availability of active sites in the solid-state configuration [50]. Consequently, to achieve effective microbial inhibition in food packaging applications, chitosan-based films are often functionalized with additional antimicrobial agents, such as essential oils, plant extracts, or metal nanoparticles [51]. These additives enhance the bioactivity of the films by compensating for the loss of native antimicrobial function upon film formation, thereby extending shelf life and improving food safety [52]. In the context of food packaging, chitosan has been extensively utilized to develop biodegradable films and coatings. These materials serve to extend the shelf life of food products by reducing moisture loss, inhibiting microbial contamination, and providing a physical barrier against external damage [51]. Moreover, chitosan is employed as an edible coating for fresh produce such as fruits and vegetables, offering an added layer of protection that aligns with consumer demand for safe, eco-friendly packaging. Its safety profile and functional versatility make chitosan a promising candidate for sustainable food packaging applications [51].

Nevertheless, chitosan films are not devoid of limitations. One significant challenge lies in their brittleness, which can affect handling and mechanical stability. Additionally, chitosan is not as widely available as some other biopolymers, which may restrict its scalability and commercial adoption. Despite these constraints, chitosan continues to receive attention as a viable material for biodegradable packaging. Current research efforts are directed toward the development of advanced fabrication techniques and composite formulations aimed at enhancing the flexibility, strength, and barrier performance of chitosan-based films, thereby broadening their applicability in the packaging industry.

### 2.5. Alginate

Alginate is an anionic polysaccharide that occurs naturally in brown seaweeds and certain microbial species. Its molecular structure is composed of varying proportions and arrangements of α-L-guluronic acid (G) and (1–4)-linked β-D-mannuronic acid (M) residues. Depending on the biological source, the linear copolymer may present as homopolymeric sequences (MM or GG blocks) or as heteropolymeric segments (MG blocks). Of particular interest are the GG blocks, which exhibit a high binding affinity for divalent cations such as calcium, thereby facilitating the formation of stable gels [53]. This unique gel-forming capacity, in combination with its non-toxicity and biodegradability, renders alginate a highly promising material for developing biodegradable films for food packaging applications [53].

The functional properties of alginate can be tailored by modifying the extraction and processing conditions. Specifically, alginate films can be engineered to achieve controlled mechanical strength and sustained release of active compounds by incorporating calcium ions to induce gelation. The primary sources of alginate include brown seaweeds such as *Macrocystis pyrifera*, *Ascophyllum nodosum*, *Laminaria hyperborea*, and *Laminaria digitata*, with the composition and physicochemical attributes of the extracted alginate being influenced by the species type and its growing environment [54]. In addition to macroalgal sources, alginate can also be biosynthesized by microorganisms such as *Azotobacter vinelandii* and *Pseudomonas aeruginosa* [53]. Notably, alginate derived from bacterial sources differs structurally and compositionally from that of algal origin, resulting in distinct functional characteristics [54].

The use of alginate in biodegradable films for packaging agricultural produce offers numerous advantages. It is environmentally benign, naturally decomposable, and safe for contact with food. Alginate-based films provide an effective barrier against oxygen and moisture, thereby prolonging the freshness of perishable products. Furthermore, its gel-forming ability can be exploited to incorporate active compounds such as antioxidants or antimicrobials into the film matrix, enabling controlled-release mechanisms that contribute to extended shelf life and improved product safety [54,55].

### 2.6. Other Polysaccharides

Pullulan consists of maltotriose units connected by α-1,6-glycosidic linkages and is a water-soluble polysaccharide. The fungal organism *Aureobasidium pullulans* is responsible for its extracellular production. As an alternative to synthetic polymers, pullulan films are interesting for food packaging because of their features such as high tensile strength, flexibility, and oxygen barrier qualities. In addition to their use in food packaging, pullulan films are also being investigated for potential applications in pharmaceuticals and cosmetics [55]. The bacteria *Xanthomonas campestris* produces xanthan gum, a high-molecular-weight polysaccharide that is anionic [56]. Trisaccharide side chains and a cellulose backbone provide it a complicated branching structure [56]. The films made by Xanthan Gum are famously stable throughout a broad temperature and pH range, very elastic, and very viscous [56]. They have a wide range of potential uses in both food and non-food industries due to their effectiveness as emulsifiers and stabilizers [57].

Several other polysaccharides, such as dextran, gellan gum, and curdlan, have also been investigated for their potential use in biodegradable films. These polysaccharides exhibit desirable properties such as compatibility with biological systems, the ability to naturally decompose, and strong film-forming characteristics [58,59]. They can be used alone or in combination with other polymers to create films with tailored properties for specific applications [58,59]. The use of these polysaccharides in biodegradable films is an area of ongoing research, and they hold promise for a wide range of applications in the food and non-food industries. Table 1 summarizes the sources and applications of various packaging films fabricated from different polysaccharides.

## 3. Enhancements in Carbohydrate-Based Bio-Film Fabrication and Characterization

Carbohydrate-based biofilms are typically prepared using a variety of methods, including solution casting, extrusion, electrospinning, layer-by-layer assembly, 3D printing, etc. [21,68]. Solution casting is the most common method for preparing carbohydrate-based films [69]. A solution of the carbohydrate, often dissolved in water or an organic solvent, is poured onto a suitable substrate (e.g., glass, Teflon). The solvent is then allowed to evaporate, leaving behind the desired film [69]. The film’s properties can be tailored by controlling factors such as the concentration of the solution, the rate of evaporation, the substrate used, and adding plasticizers, fillers, or cross-linking agents into the solution [69]. In the extrusion method, a carbohydrate-based material, often in the form of a melt or paste, is forced through a die to form a film. This technique is particularly useful for producing films with specific shapes or textures [70]. The extrusion process can be combined with other techniques, such as blowing or calendaring, to further modify the film’s properties [71]. Electrospinning involves applying a high voltage to a polymer solution or melt, causing the material to form fibers that are collected on a target. The resulting nonwoven fabric can be used to create films with unique properties, such as high surface area and porosity [72]. Electrospinning is particularly suitable for producing films from carbohydrate-based materials that are difficult to process using other methods. Layer-by-layer assembly involves sequentially depositing alternating layers of oppositely charged polyelectrolytes onto a substrate [73]. Three-dimensional printing allows for the fabrication of complex structures with controlled porosity and thickness by depositing carbohydrate-based materials layer by layer [74]. The spray coating method involves spraying a solution or suspension of the carbohydrate onto a substrate, resulting in a film with a uniform thickness [75].

The type of carbohydrate used can significantly influence the properties of the resulting film. For example, cellulose-based films tend to be strong and tough, while starch-based films are often more flexible and biodegradable [76,77]. Starch and pectin are generally soluble in water due to their hydrophilic hydroxyl and carboxyl groups, allowing easy preparation via aqueous solution casting. In contrast, cellulose, despite being a major natural polymer, is insoluble in water and most organic solvents due to its extensive hydrogen bonding and crystalline regions [35,42]. To overcome this, film formation from cellulose typically requires its conversion into derivatives (e.g., methylcellulose, carboxymethyl cellulose) or dissolution in specialized solvents such as ionic liquids or deep eutectic solvents [46]. Similarly, chitosan is soluble only in dilute acidic aqueous media owing to the protonation of its amino groups, while alginate requires ionic cross-linking with divalent cations (e.g., Ca^2+^) to form stable films [28,48]. These distinctions are critical for selecting the appropriate film fabrication method, whether casting, extrusion, or electrospinning, and must be considered when designing polysaccharide-based biodegradable films tailored to specific packaging needs. Besides the types of carbohydrates, the concentration of the carbohydrate in the solution or melt also affects the film’s properties. Higher concentrations typically lead to thicker, denser films, while lower concentrations result in thinner, more porous films [68]. The incorporation of functional materials like lignin into carbohydrate-based films can improve their mechanical properties, thermal stability, barrier properties, and biodegradability of the films [78]. Additionally, incorporating antioxidants can help prevent the degradation of carbohydrate-based films, thereby improving their shelf life and stability [79]. Adding antimicrobial agents can help protect the films from microbial contamination and extend their shelf life [80]. Plasticizers, including glycerol, sorbitol, and polyethylene glycol, are added to carbohydrate-based films to enhance flexibility and diminish brittleness. The kind and quality of plasticizer employed can significantly influence the film’s mechanical characteristics and water vapour permeability [81]. The processing conditions, such as temperature, time, and drying rate, can also influence the properties of carbohydrate-based films. For example, higher temperatures can accelerate the evaporation of the solvent and reduce the film’s moisture content, while longer drying times can improve the film’s mechanical properties [82]. By carefully selecting the type and concentration of carbohydrates, the use of functional materials, the choice of plasticizer, and the processing conditions, one may customize the characteristics of carbohydrate-based films to fulfill specific applications and needs.

Several important film properties are relevant to packaging applications, including mechanical strength (such as tensile strength and elongation), barrier performance (against oxygen and water vapour), and optical characteristics (such as transparency and haze), as well as the potential for natural decomposition and suitability for composting. The mechanical properties include tensile strength, which measures the utmost stress a film may endure prior to failure under tensile load. Increased tensile strength indicates better resistance to tearing and puncture [83]. Elongation at break measures the maximum amount a film can stretch before breaking. Higher elongation indicates better flexibility and resistance to cracking [84]. Tear strength measures the force required to propagate a tear in the film. Higher tear strength indicates better resistance to tearing and puncture [83]. Puncture resistance measures the force required to puncture the film with a sharp object. Higher puncture resistance indicates better protection against damage [85]. Similarly, barrier properties, such as oxygen permeability, measure the rate at which oxygen diffuses through the film. Lower oxygen permeability is desirable for packaging products that are sensitive to oxidation, such as fruits, vegetables, and meat [86]. Water vapor permeability measures the rate at which water vapor diffuses through the film. Lower water vapor permeability is desirable for packaging products that require protection from moisture, such as dried foods and pharmaceuticals [87]. Aroma barrier properties measure the film’s ability to prevent the diffusion of aromas. Higher aroma barrier properties are important for packaging products with strong odors, such as coffee and spices [88]. In optical properties, transparency measures the ability of the film to transmit visible light. Higher transparency is desirable for packaging products that need to be visible to consumers, such as fruits and vegetables [89]. Haze measures the amount of light scattered by the film. Lower haze indicates a clearer and more transparent film [90]. Gloss measures the amount of light reflected from the film’s surface. Higher gloss indicates a shinier and more attractive film [91]. Biodegradability of the film implies that the film can decompose naturally into harmless substances by microorganisms. Higher biodegradability is desirable for reducing environmental impact [92]. Compostability refers to the ability of the film to decompose in a compost environment and become part of the soil. Compostable films can be safely disposed of in compost bins [93]. However, considering all the properties discussed above, the specific film properties required for packaging applications will vary depending on the product being packaged and the desired performance characteristics. For example, packaging for fresh produce may prioritize oxygen and water vapor barrier properties, while packaging for dry goods may focus on moisture barrier properties. By understanding the key film properties and their relevance to packaging applications, manufacturers can select or develop films that meet the specific needs of their products. Table 2 represents a comparative overview of key fabrication techniques employed in the production of carbohydrate-based biodegradable films. It outlines the distinctive film characteristics, compatible materials, and common enhancement strategies associated with each method, offering insights into process-specific advantages and functional optimization.

## 4. Applications in Agricultural Commodity Packaging

Table 3 presents a comparative and analytical summary of the application of polysaccharide-based biodegradable films for packaging a range of agricultural commodities. This tabular analysis is intended to provide a clearer understanding of how various naturally derived polymers, such as pectin, starch, cellulose, chitosan, alginate, pullulan, and xanthan gum, can be strategically employed based on the unique preservation needs of different food categories. The commodities covered include fresh produce (fruits and vegetables), animal-derived products (meat, poultry, dairy), cereals and grains, nuts and seeds, dried fruits, and oil-based products. For each commodity type, the table identifies the most commonly used polysaccharide films and their primary functional roles, such as acting as barriers to moisture, oxygen, and microbial contamination. These functions directly influence the extension of shelf life, maintenance of food quality, and reduction of spoilage. For example, chitosan-based films are extensively used in meat and poultry packaging due to their strong antimicrobial activity, while cellulose and alginate are preferred for dry commodities like cereals and grains for their effective moisture control.

The table also delineates packaging-specific requirements, such as transparency, flexibility, oil resistance, or mechanical strength, and matches these with the material’s performance characteristics. Importantly, the limitations associated with each film type in specific applications are critically summarized. These include common challenges such as hydrophilicity, brittleness under low humidity, incompatibility with high-fat contents, and degradation under fluctuating moisture conditions. To address these limitations, the table outlines potential improvement strategies, including the incorporation of nanofillers, cross-linking agents, hydrophobic additives, multilayer structures, or blending with complementary biopolymers. This comprehensive yet concise format enables a targeted evaluation of the suitability and adaptability of polysaccharide films for specific packaging scenarios, thereby offering valuable insights for material optimization in sustainable packaging systems.

## 5. Advantages of Carbohydrate-Based Bio-Degradable Films

Carbohydrate-based biodegradable films offer a range of environmentally beneficial and functionally valuable characteristics, many of which are closely tied to the unique physicochemical properties of the specific polysaccharide used [19,21]. These biofilms not only reduce dependency on petroleum-based polymers but also offer customizable features such as barrier properties, biodegradability, and compatibility with active packaging components. Among these, starch-based films are particularly notable for their abundance, low cost, and strong film-forming capability [23]. Their effectiveness as oxygen barriers under low-humidity conditions makes them suitable for packaging dry food products [23]. However, their intrinsic hydrophilicity limits their performance under high-moisture environments [39]. These limitations can be mitigated through physical blending with hydrophobic materials or chemical modification techniques to improve water resistance [23,39,41]. In contrast, cellulose and its derivatives such as carboxymethyl cellulose and methylcellulose provide excellent mechanical strength, high transparency, and structural integrity [46]. Owing to its semi-crystalline structure and extensive hydrogen bonding, cellulose exhibits superior gas barrier properties [46,77]. Although native cellulose is insoluble in water, its derivatives are more suitable for solution processing. The inherent rigidity of cellulose films makes them ideal for packaging applications requiring durability and strength. Chitosan-based films, derived from chitin, present another significant advantage—namely, their potent antimicrobial and antifungal activities [26]. These attributes make chitosan a preferred choice for packaging perishable food items such as meats and seafood [26]. Additionally, chitosan films form easily in acidic aqueous media and provide moderate mechanical strength. Nonetheless, their sensitivity to moisture can restrict their use in high-humidity environments unless reinforced with hydrophobic polymers or nanomaterials [31]. Similarly, alginate-based films, extracted from brown algae, are valued for their biocompatibility, moisture retention, and gel-forming capabilities [28]. These properties enable their use in packaging of products that benefit from a controlled release environment. However, their mechanical and water resistance properties require enhancement, often achieved through ionic cross-linking using divalent cations such as calcium [28]. Completing the spectrum, pectin-based films offer good flexibility, optical clarity, and intrinsic antioxidant potential, making them suitable for wrapping fresh produce and products susceptible to oxidation [30,35]. Despite their moderate mechanical performance, pectin films exhibit strong film-forming behavior and can be effectively combined with other polysaccharides or natural additives to enhance stability and functional properties.

Collectively, these polysaccharide-based films demonstrate distinct advantages tailored to specific food packaging requirements. Their diversity underscores the importance of a targeted, property-driven approach in selecting suitable biopolymers to meet application-specific challenges in sustainable packaging design. Moreover, carbohydrate-based biodegradable films are developing as a sustainable and eco-friendly substitute for traditional petroleum-based polymers in the food packaging sector. These films, sourced from renewable materials like starch, cellulose, and chitosan, provide several benefits that tackle the escalating issues of plastic waste and pollution.

### 5.1. Environmental Benefits

A primary benefit of carbohydrate-based films is their biodegradability. These films have the ability to completely break down into carbon dioxide, water, and biomass under composting conditions [55,101], contributing to a more sustainable end-of-life disposal. This biodegradability helps reduce the accumulation of plastic waste in landfills and the environment, mitigating the negative impacts on wildlife and ecosystems. It is estimated that more than 300 million tons of plastic garbage are produced worldwide each year, with a significant proportion originating from food packaging [104]. This waste contributes to environmental pollution, harms wildlife, and depletes natural resources. In contrast, carbohydrate-based films offer a sustainable solution by reducing plastic waste and minimizing reliance on fossil fuels. Life cycle assessment (LCA) studies have shown that carbohydrate-based films have a lower environmental impact compared to conventional plastics [104]. The synthesis and degradation cycles of biopolymers are frequently carbon neutral or negative, hence diminishing greenhouse gas emissions and reliance on fossil fuels [105]. LCA also considers factors such as water use, energy consumption, and waste generation throughout the entire life cycle of the packaging material.

### 5.2. Economic Benefits

Even though the initial cost of biopolymer may be higher than that of traditional plastics, cost has been coming down. Innovations in production and processing techniques, along with improved technology, allow companies to make them cheaper [106]; using waste biomass from sales of produce at agro-food industries such as mango seeds or spent coffee beans further reduces the cost of producing carbohydrate-based films [107]. As the market grows and economies of scale come into play, carbohydrate-based films are ever more cost-effective. As a result of consumer demand for green products and an increase in sustainable products, the global bioplastics market is quickly growing [108]. The bioplastics market is expected to produce over 15% more every year for the coming years. By 2022, it will be made of some 2.4 million tons a year [109]. This expanding market offers a significant business opportunity to carbohydrate film in various food packaging applications.

### 5.3. Technical Benefits

Carbohydrate-based films have several technical benefits that render them appropriate for various food packaging applications. They exhibit exceptional compatibility with many agricultural commodities, rendering them optimal for the packaging of fresh fruit, meat, dairy products, baked goods, and other food items [110]. They can be tailored to specific food packaging requirements by adjusting their permeability to gases, moisture, and flavors. Additionally, carbohydrate-based films can enhance the quality and safety of food items by serving as a barrier against external pollutants, including gases, light, dust and microbes [111]. They can also be incorporated with active ingredients like antioxidants and antimicrobial agents to further enhance food preservation [112].

The versatility of carbohydrate-based films is another technical advantage. They can be processed into various forms, including films, trays, bottles, and other packaging formats [113]. They can be made by different methods, such as extrusion, casting, and thermoforming, to meet specific packaging needs. Natural carbohydrate-based polymers are non-toxic and exhibit minimal interaction with foods, ensuring food safety and quality [114]. Many carbohydrate-based films are suitable for industrial composting, and certain types, such as PLA, may also undergo mechanical recycling [115]. This further enhances their sustainability and reduces their environmental impact.

In conclusion, carbohydrate-based biodegradable films offer a multitude of environmental, economic, and technical benefits. Their biodegradability, compatibility with various food items, and potential for cost-effectiveness make them attractive alternatives to conventional plastics. As research and development continue to advance biopolymer technologies, these films are poised to play an even greater role in creating sustainable and eco-friendly food packaging solutions.

**Figure 3:**
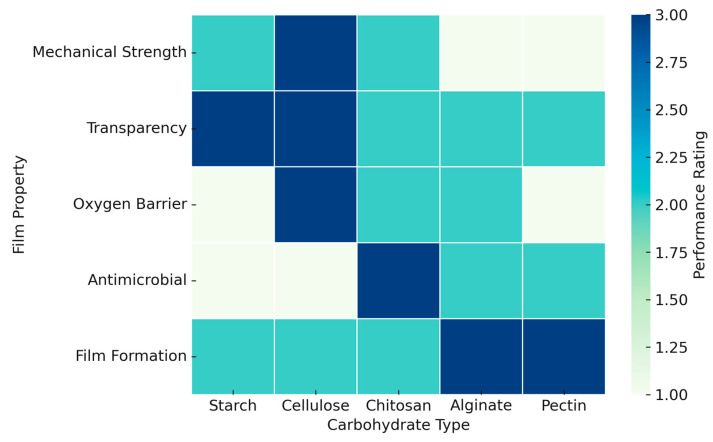
Advantages of different carbohydrates-based food packaging films [116,117,118].

Figure 3 provides a comparative overview of key functional properties exhibited by different carbohydrate-based biodegradable films. The selected polysaccharides—starch, cellulose, chitosan, alginate, and pectin—are evaluated based on parameters such as oxygen barrier, mechanical strength, antimicrobial activity, water resistance, transparency, and film-forming ability. This matrix highlights the distinct strengths and limitations of each material, offering a practical guide for material selection in packaging design. The qualitative rankings are synthesized from recent literature and experimental findings.

## 6. Challenges and Future Perspectives

### 6.1. Economic Challenges and Cost Competitiveness

The main challenge is the cost-effectiveness compared to traditional plastics. Traditional plastic films, such as polyethylene and polypropylene, are inexpensive due to established large-scale production and widespread availability of petroleum-based raw materials [119]. In contrast, carbohydrate-based films are expensive due to limited production capacity and higher processing expenses [120]. The challenges may be faced as raw materials (e.g., starch, cellulose) may be more expensive than petrochemical-derived polymers [121]. Additional processing steps (e.g., blending, plasticizing, and reinforcing) increase manufacturing costs. Therefore, investment should be made in technology to improve the efficiency of carbohydrate film production. Cost-effective agricultural waste-based feedstocks should be developed (e.g., corn husks and cassava waste) to reduce raw material costs. Government subsidies and tax incentives should be incorporated for biodegradable packaging to level the playing field against traditional plastics.

### 6.2. Technological Limitations and Industrial Scale-Up

While carbohydrate-based films show promise in lab-scale or niche applications, scaling up production to industrial levels is difficult. Conventional plastic production is highly optimized, while large volumes of carbohydrate films still face technological and logistical barriers. The main issues are that the equipment and processes for mass production of these films (e.g., extrusion, casting) are not as advanced as those for synthetic polymers [122]. As there are limited infrastructure and facilities for producing biodegradable films on a global scale, there is an inconsistent quality across large batches due to the variability in natural carbohydrate sources [123]. Investment in research and development to scale up production technologies, such as roll-to-roll film production and continuous casting techniques, could be a potential solution. Also, ensuring the standardization of carbohydrate raw materials and processing methods to ensure consistent film properties at scale. Additionally, considering public–private partnerships to build larger production facilities focused on biopolymer films would also help.

### 6.3. Material Performance and Property Enhancement

Challenges are also faced while improving certain film properties such as water resistance, flexibility, and printability. Carbohydrate-based films often lack the robustness required for certain packaging applications. Properties like water resistance, flexibility, durability, and printability are not yet comparable to synthetic films. Carbohydrate films tend to absorb moisture, leading to reduced strength and structural integrity [124]. Poor mechanical properties, such as brittleness, especially in dry environments, reduce the usability of these films in packaging [125]. A limited ability to print or apply high-quality graphics on carbohydrate-based films compared to plastics can negatively impact branding and labeling [126]. Therefore, developing composite films by combining carbohydrate materials with other biopolymers, lipids, or waxes will improve water resistance and flexibility [127]. The use of plasticizers like glycerol can improve film flexibility without compromising biodegradability [128]. Implementing advanced surface treatments or coatings can enhance printability and make carbohydrate films more appealing for commercial use [129].

### 6.4. Consumer Acceptance and Market Adoption

Despite growing environmental awareness, consumers may be hesitant to adopt carbohydrate-based films due to concerns about their appearance, durability, or cost. Many consumers are unfamiliar with carbohydrate-based films and may question their effectiveness compared to conventional plastics. It is perceived that biodegradable packaging may lead to shorter shelf life or diminished product protection, and biodegradable and eco-friendly packaging options are often priced higher, which may deter cost-conscious consumers [130]. However, increasing public awareness and education on the environmental benefits of biodegradable packaging will drive consumer demand [131]. More work should be put into product design and marketing to make carbohydrate-based packaging more visually appealing and to showcase its benefits. Additionally, collaboration with retailers to incentivize the use of biodegradable films, including trial runs and price subsidies, will make eco-friendly packaging more accessible.

### 6.5. Authors’ Hypothesis and Innovation Pathways

The authors hypothesize that the key to overcoming these challenges lies in the development of hybrid, multifunctional films that incorporate natural nanomaterials, cross-linking agents, and bioactive compounds to enhance mechanical, barrier, and active functionalities without compromising biodegradability. Advancements in green processing technologies, such as reactive extrusion, supercritical fluid processing, and solvent-free methods, could also significantly lower production costs and improve scalability. Additionally, establishing standard testing protocols, scaling pilot manufacturing, and implementing circular economy strategies, such as composting infrastructure and extended producer responsibility, will be crucial for transitioning to bio-based packaging systems. Future research should focus on life cycle assessment, end-of-life analysis, and integration of biosensors for smart packaging applications. Altogether, the shift from synthetic plastics to biodegradable packaging requires a multidisciplinary approach that leverages materials science, industrial engineering, policy innovation, and consumer engagement.

### 6.6. Future Research Directions

Moreover, future research should include the development of novel carbohydrate sources and blends. This includes exploring alternative carbohydrate sources from non-traditional crops or agricultural by-products (e.g., cassava, algae, seaweed) to diversify the supply chain and reduce reliance on common sources like corn and potatoes. Additionally, investigating underutilized carbohydrates such as hemicellulose, pectin, and inulin for potential applications in biodegradable packaging should also be encouraged. The development of composite films by blending carbohydrates with other biopolymers (e.g., proteins, polyhydroxyalkanoates) or nanomaterials (e.g., nanocellulose, starch nanocrystals) will enhance mechanical, barrier, and functional properties. These blended films can offer improved functionality (e.g., flexibility, durability, and moisture resistance) while maintaining biodegradability and providing access to a broader range of raw materials that may improve the cost-efficiency, performance, and sustainability of carbohydrate-based films. The research focus should be based on improving existing production methods, such as extrusion, casting, and electrospinning, to enhance the mechanical and barrier properties of carbohydrate-based films, along with exploring the use of advanced techniques like solvent casting, electrospinning, and layer-by-layer deposition to produce films with more precise control over thickness and composition. An investigation should be conducted into bio-based plasticizers and cross-linking agents to improve the flexibility, water resistance, and stability of carbohydrate films. This will enhance the scalability of production methods, making these films more viable for industrial applications and optimizing film properties, including better moisture barriers, improved mechanical strength, and better compatibility with various food products.

### 6.7. Next-Generation Packaging and Regulatory Needs

The potential future perspective also includes research on the next generation of packaging materials, such as smart packaging, active packaging, and edible packaging. Smart packaging integrates sensors or indicators into carbohydrate-based films to monitor the quality and safety of food products (e.g., freshness, spoilage, temperature changes). These films could communicate real-time information to consumers or supply chain managers, reducing food waste and ensuring product quality. Active packaging develops films that can interact with the packaged product or the environment to continue shelf life by releasing or absorbing certain compounds (e.g., antimicrobial agents, oxygen scavengers, ethylene absorbers), which actively prevent microbial growth, inhibit oxidation, or control ripening processes, which would enhance food safety and longevity [79]. Edible packaging creates edible carbohydrate-based films that act as a protective barrier but can be safely consumed with the product, eliminating packaging waste by making the film part of the product, which is especially useful for single-serve items (e.g., fruits, vegetables, snack bars) [118].

The development of global standards for biodegradable packaging materials, including carbohydrate-based films, to ensure consistency in material properties, biodegradability, and food safety, will improve regulatory and standardization issues. There should be clarity on addressing regulatory challenges regarding the use of bio-based materials, including what constitutes “biodegradable” and “compostable” under different conditions. Collaboration with regulatory bodies (e.g., FDA, EFSA) will ensure that active or edible packaging is safe for human consumption and does not interact negatively with food products. There should be clear guidelines and certifications for companies producing carbohydrate-based packaging that can increase consumer and industry trust. Additionally, streamlining the approval process for novel packaging materials will accelerate their market entry and adoption. Moreover, developing new materials and fabrication methods, future perspectives should also address the fate of carbohydrate-based films after their intended use. While these materials are biodegradable, recycling and valorization strategies for films withdrawn from use can further enhance their sustainability. Biodegradable films can be subjected to industrial composting, enzymatic hydrolysis, or anaerobic digestion, leading to the production of nutrient-rich compost or biogas, thereby recovering energy and nutrients from waste streams. Furthermore, research is advancing toward the recovery of monomeric sugars or bio-based chemicals through selective depolymerization or microbial fermentation of spent films. These end-of-life pathways not only reduce environmental impact but also contribute to a more circular material economy. Integration of recycling or recovery technologies with biodegradable film production systems can thus play a pivotal role in minimizing resource loss and closing the loop for biopolymer-based packaging.

## 7. Conclusions

Carbohydrate-based biodegradable films derived from natural polysaccharides such as starch, cellulose, chitosan, pectin, and alginate offer substantial promise as eco-friendly alternatives to petroleum-based packaging materials. These biopolymers are renewable, biodegradable, and safe for food contact applications. Their film-forming potential, combined with desirable properties such as oxygen barrier, transparency, and antimicrobial activity, highlights their suitability for replacing conventional plastics in packaging applications. However, the widespread adoption of these materials is still limited by several technical and economic barriers, including high water sensitivity, limited mechanical robustness, elevated production costs, and difficulties in achieving industrial scalability. To address these challenges, recent research has focused on composite and hybrid film development, incorporation of plasticizers and cross-linkers, and nanomaterial reinforcement to enhance flexibility, water resistance, and durability without compromising biodegradability. Equally important is the optimization of film fabrication techniques, which play a crucial role in determining the final performance and processability of biodegradable films. Techniques such as solution casting, extrusion, electrospinning, and layer-by-layer assembly offer distinct advantages depending on the application scale and film requirements. Efforts are underway to transition from laboratory-scale methods to more scalable processes such as roll-to-roll coating and continuous casting, which can help reduce costs and improve batch consistency. The integration of bioactive compounds and surface treatments during fabrication further expands the functional capabilities of these films. In terms of environmental impact, the use of biodegradable carbohydrate-based films presents a meaningful opportunity to reduce the overall carbon footprint associated with traditional plastics. While synthetic polymers benefit from established large-scale manufacturing and low initial costs, their non-biodegradable nature results in significant post-consumer waste and long-term ecological harm. Biodegradable films, though initially more resource-intensive, offer end-of-life benefits such as compostability and reduced emissions, making them favorable within circular economy frameworks. Overcoming the limitations of both materials and processing technologies requires a systems-level approach, one that incorporates material innovation, fabrication efficiency, and lifecycle sustainability. As the field advances, research should prioritize the standardization of bio-based raw materials, development of multifunctional films through optimized fabrication, and establishment of scalable, low-energy manufacturing protocols. Ultimately, coupling material advancements with supportive policy, infrastructure development, and consumer awareness will be essential to achieving the transition from conventional plastic to biodegradable packaging on a global scale.

**Overall Summary**

- Carbohydrate polymers offer eco-friendly alternatives to plastic food packaging.
- Each biopolymer shows unique strengths and limits for specific food applications.
- Innovative additives improve film flexibility, barrier function, and durability.
- Antimicrobial films can extend shelf life, especially when enhanced with agents.
  A shift to biofilms supports the circular economy and reduces plastic pollution.

## Figures and Tables

**Table 1:** Food packaging application of various biopolymer-based films.

Film Type	Properties	Food Products Packaged	Packaging Functionality	Challenges	Sources	Film Fabrication Methods	References
Pectin-based	Biocompatible, gelling ability, antioxidant-rich	Cheese, meats, seafood	Barrier to microbes and gases	Low mechanical strength, water sensitivity	Apple pomace, citrus peels	Solution casting, extrusion	[35,36]
Starch-based	Abundant, low-cost, flexible, biodegradable	Tomatoes, chicken, bakery	Moisture retention, transparency	High water vapor permeability	Corn, potato, cassava	Extrusion, thermo-compression	[39]
Cellulose-based	High mechanical strength, transparency, oxygen barrier	Fruits, vegetables, cereals	Strength, gas permeability control	Hydrophilic, poor thermal resistance	Wood pulp, cotton, agro-residues	Solvent casting, roll coating	[46]
Chitosan-based	Antimicrobial, biodegradable, edible	Poultry, cheese, berries	Antimicrobial action, preservation	Brittle at neutral pH, limited flexibility	Shrimp shells, insect chitin	Casting, layer-by-layer coating	[51]
Pullulan-based	Transparent, flexible, good oxygen barrier	Dried snacks, bakery items	Oxygen barrier, flexibility	High cost, water sensitivity	Fungal fermentation (*A. pullulans*)	Casting, spray drying	[55,60]
Xanthan gum-based	High viscosity, stable under pH and temperature variation	Sauces, dressings, jellies	Thickening and emulsifying	Poor mechanical strength, needs blends	*Xanthomonas campestris*	Solution casting, blending	[61,62]
Alginate-based	Film-forming, biocompatible, ionically cross-linkable	Oils, fruits, nutraceuticals	Moisture and aroma retention	Weak moisture barrier, complex gelation	Brown seaweed, bacterial alginate	Ionic gelation, solvent evaporation	[54,55,63]
Composite films	Tailored barrier and mechanical properties via blending	Meat, multilayer packaging	Improved durability and barrier	Formulation complexity, regulatory limits	Blends of biopolymers, nanomaterials	Extrusion, lamination	[64,65,66,67]
Gellan gum-based	Good thermal stability, forms firm gels	Meat gels, dairy, snacks	Firm texture, thermal control	Extraction complexity, high cost	Bacterial fermentation (*Sphingomonas* spp.)	Calcium cross-linking, drying	[58]
Curdlan-based	Forms thermally reversible gels, biodegradable	Soy-based foods, gluten-free items	Texturization, preservation	Thermal instability in some blends	Alkaline treatment of microbial polysaccharides	Heat-induced gelation, film pressing	[59]

**Table 2:** Fabrication methods for carbohydrate-based biofilms.

Fabrication Technique	Film Characteristics	Material Compatibility	Enhancement Strategies	References
Solution Casting	Uniform thickness, good surface finish, suitable for lab-scale films	Starch, pectin, cellulose derivatives, xanthan gum	Plasticizers, nanofillers, cross-linkers, active agents	[69]
Extrusion	Continuous film production, specific shapes/textures, suitable for industrial scale	Starch, PLA blends, thermoplastic derivatives	Blending, nanocomposites, multilayer film integration	[70,71]
Electrospinning	High surface area, porous structure, ideal for functionalized films	Chitosan, cellulose nanofibers, PVA blends	Encapsulation of active compounds, nanofiber reinforcement	[72]
Layer-by-Layer Assembly	Nano-to-microscale multilayers, controlled composition, high barrier properties	Charged polysaccharides (e.g., chitosan, alginate)	Cross-linking, interfacial adsorption, layer functionalization	[73]
3D Printing	Complex geometries, custom shapes and porosity, emerging technique	Starch-based hydrogels, alginate composites	Functional additives, multilayer constructs	[74]
Spray Coating	Thin and uniform coatings, scalable, low waste	Starch, pectin, and soluble carbohydrate solutions	Active ingredients, surfactants, stabilizers	[75]

**Table 3:** Various applications of carbohydrate-based film in packaging agricultural commodities.

Commodity	Film Type	Functional Role	Application Example	Packaging Requirement	Technical Challenges	Suggested Improvements	References
Fruits and Vegetables	Pectin, Starch, Chitosan	Gas and moisture barrier, antimicrobial	Pectin coating for apples	Breathability, light weight, transparency	Low mechanical strength, moisture sensitivity	Blend with lipids, add cellulose or nanofillers	[87,94]
Meat and Poultry	Chitosan, Composite Films	Oxidation and microbial inhibition	Chitosan wrap for beef/chicken	Puncture resistance, moisture retention	Film degradation in humid conditions	Composite films with proteins, cross-linking	[95,96]
Dairy Products	Starch, Chitosan	Moisture barrier, mold inhibition	Starch film for cheese aging	Control of humidity and microbial growth	Fat interaction reduces film integrity	Add hydrophobic agents or protein blends	[97,98]
Cereals and Grains	Cellulose, Alginate	Humidity and oxygen barrier	Cellulose wrap for rice	Stable moisture barrier under dry conditions	Brittleness in low RH environments	Use of plasticizers, multilayer structures	[99,100]
Nuts and Seeds	Pectin, Pullulan	Moisture/O_2_ barrier, oil retention	Pullulan film on roasted nuts	Aroma protection, oil migration resistance	Oil absorption reduces strength	Coatings with waxes, lamination	[27]
Dried Fruits	Xanthan gum, Alginate	Preservation of softness and appearance	Alginate-coated figs	Transparency, oxidation resistance	Cracking under storage stress	Incorporate glycerol or glycerides	[27]
Grain Snacks	Starch, Chitosan	Barrier to gas and texture degradation	Starch wrap for puffed snacks	Lightweight, rigid films	Poor tensile strength	Reinforce with nanocellulose	[101,102]
Vegetable Oils	Alginate, Composite	Spill resistance, oil absorption control	Alginate sachets	Hydrophobic and oxidation barriers	Oil permeability over time	Layered systems, surface coating	[103]

## Data Availability

All the data are presented within this review article.
